# Extensive Metabolic Remodeling Differentiates Non-pathogenic and Pathogenic Growth Forms of the Dimorphic Pathogen *Talaromyces marneffei*

**DOI:** 10.3389/fcimb.2017.00368

**Published:** 2017-08-17

**Authors:** Shivani Pasricha, James I. MacRae, Hwa H. Chua, Jenny Chambers, Kylie J. Boyce, Malcolm J. McConville, Alex Andrianopoulos

**Affiliations:** ^1^Genetics, Genomics and Systems Biology, School of BioSciences, University of Melbourne Parkville, VIC, Australia; ^2^Department of Biochemistry and Molecular Biology, Bio21 Institute of Molecular Science and Biotechnology, University of Melbourne Parkville, VIC, Australia

**Keywords:** *Talaromyces marneffei*, central carbon metabolism, metabolomics, nutrient acquisition, pathogenic fungi, dimorphic switching

## Abstract

Fungal infections are an increasing public health problem, particularly in immunocompromised individuals. While these pathogenic fungi show polyphyletic origins with closely related non-pathogenic species, many undergo morphological transitions to produce pathogenic cell types that are associated with increased virulence. However, the characteristics of these pathogenic cells that contribute to virulence are poorly defined. *Talaromyces marneffei* grows as a non-pathogenic hyphal form at 25°C but undergoes a dimorphic transition to a pathogenic yeast form at 37°C *in vitro* and following inhalation of asexual conidia by a host. Here we show that this transition is associated with major changes in central carbon metabolism, and that these changes are correlated with increased virulence of the yeast form. Comprehensive metabolite profiling and ^13^C-labeling studies showed that hyphal cells exhibited very active glycolytic metabolism and contain low levels of internal carbohydrate reserves. In contrast, yeast cells fully catabolized glucose in the mitochondrial TCA cycle, and store excess glucose in large intracellular pools of trehalose and mannitol. Inhibition of the yeast TCA cycle inhibited replication in culture and in host cells. Yeast, but not hyphae, were also able to use *myo*-inositol and amino acids as secondary carbon sources, which may support their survival in host macrophages. These analyses suggest that *T. marneffei* yeast cells exhibit a more efficient oxidative metabolism and are capable of utilizing a diverse range of carbon sources, which contributes to their virulence in animal tissues, highlighting the importance of dimorphic switching in pathogenic yeast.

## Introduction

Pathogenic microbes frequently have to adjust their cellular metabolism in order to exploit particular nutrient conditions in the infected host tissues and/or to effectively respond to physical or microbiocidal stresses imposed on them by the host (Rohmer et al., 2011). For many microbes, including an increasing number of dimorphic fungal pathogens such as the opportunistic pathogens *Talaromyces marneffei, Histoplasma capsulatum, Blastomyces dermatitidis* and others, metabolic adaptation is also required to maintain a saprophytic lifestyle in the soil or in other non-human host environments (Schoberle and May, 2007; Cramer et al., 2008; Hu et al., 2008; Askew et al., 2009; Brock, 2009; Fleck et al., 2011; Han et al., 2011; Rohmer et al., 2011; Brown et al., 2014).

The opportunistic fungal pathogen *T. marneffei* is considered the third most common pathogen in AIDS-related diseases in Northern Thailand and an important public health threat across South East Asia where it is endemic (Rongrungruang and Levitz, 1999; Walsh and Groll, 1999). In common with a number of other pathogenic fungi, *T. marneffei* undergoes a reversible morphological switch from a filamentous, multicellular (hyphal or pseudohyphal) to a unicellular (yeast) growth form. The *T. marneffei* non-pathogenic hyphae proliferate at environmental temperatures (~25°C) and can produce asexual conidia. Host innate immune cells, particularly macrophages, phagocytose inhaled conidia and it is within these host cells that they undergo the dimorphic switch to the pathogenic yeast form. The yeast cells circumvent the microbiocidal action of host macrophages and replicate within intracellular vacuoles. The dimorphic switch from the hyphal to yeast form is considered a key virulence attribute that allows this fungus to survive within this hostile host niche.

A detailed understanding of the physiological and metabolic changes that underpin the dimorphic transition and the two morphological states is important for understanding the pathogenicity of dimorphic fungi such as *T. marneffei* and developing new therapies (San-Blas et al., 2000; Gow et al., 2002; Klein and Tebbets, 2007). Toward this goal, we have undertaken the first comprehensive metabolomic analysis of hyphal and yeast cells in a dimorphic pathogen. These analyses highlight major differences in the carbon source utilization, central carbon metabolism and the accumulation of compatible solutes in each of these cell types and identify pathways that are essential for yeast growth in macrophages. These changes in cellular metabolism appear to be linked to the signals associated with the dimorphic shift and may thus pre-adapt *T. marneffei* (and potentially other dimorphic fungi) to life in the mammalian host.

## Materials and methods

### Fungal strains and growth conditions

*T. marneffei* type strain FRR2161 was provided by Dr. J. Pitt (CSIRO Food Industries, Sydney). For metabolite extraction, *T. marneffei* conidia (1 × 10^7^) were cultured in 100 mL of heart infusion (HI) medium (Oxoid). For production of yeast cells, *T. marneffei* was cultured at 37°C for 4 days. Then 10 mL of yeast cells were transferred to fresh HI medium and grown to the mid-logarithmic (mid-log) phase (20 h) prior to harvesting. For production of hyphal cells, cultures were grown at 25°C to mid-log phase (45 h) in HI medium prior to harvesting (Figure S1).

For sole carbon source growth assays, *T. marneffei* conidia (1 × 10^5^) were cultured in 20 mL of yeast nitrogen base liquid medium [supplemented with 10 mM (NH_4_)_2_SO_4_] containing sole carbon source (50 mM) glucose, fructose, *myo*-inositol, malate, proline, or alanine. Conidia were cultured at 25°C and incubated for 2 days for hyphal growth or 37°C for 4 days, followed by transfer of 2 mL culture to fresh medium for an additional 2 days of yeast cell growth. The optical density under each condition was measured using a spectrophotometer. Density measurements for hyphal and yeast conditions were normalized separately against cell density when cultured in glucose as the sole carbon source at 25 and 37°C, respectively (representing 100% growth).

For growth assays in the presence of sodium fluoroacetate (NaFAc), *T. marneffei* conidia (1 × 10^7^) were grown in 100 mL of yeast nitrogen base liquid medium containing 50 mM glucose and 10 mM (NH_4_)_2_SO_4_. Hyphal cell growth was at 25°C for 2 days. Yeast cell growth was at 37°C for 4 days followed by transfer of 10 mL of this culture to fresh medium and additional incubation for 2 days. Following the addition of 20 mM NaFAc, cultures were incubated for an additional 0, 24, or 48 h. Cells were harvested onto filter discs (Whatman) using a filter apparatus connected to a vacuum pump and pellets were dehydrated at 60°C overnight. Desiccated cells of each sample at both 25° and 37°C were measured as dry mass.

### Metabolite footprinting of hyphal and yeast cultured media using GC-MS

*T. marneffei* was grown as described above. Upon reaching mid-log growth, exogenous glucose [6 mM]_fin_ was added to the cultures and aliquots (600 μL) of cultured medium sampled hourly, transferred to 1.5 mL microfuge tubes and centrifuged (16,000 × g, 15 min, 0°C). A aliquot (10 μl) of the supernatants were analyzed by GC-MS, as described below, and the metabolite content of four technical replicates were quantified for each time point.

### U-^13^C-glucose-labeled metabolite foot-printing of hyphal and yeast cultured media using NMR

Upon reaching mid-log, U-^13^C-glucose [6 mM]_fin_ (Cambridge Isotope Laboratories) was added to the cultures. At given time points, aliquots of 600 μL of the culture medium was transferred to a 1.5 mL microfuge tubes and centrifuged at 16,000 × g (15 min, 0°C) (4 technical replicates). Supernatants (500 μL) were transferred to new microfuge tubes, prepared for NMR analysis and quantified as described previously (Lutz et al., 1996).

### Collection of medium controls and the metabolite extraction of *T. marneffei* yeast and hyphal cells

At the appropriate growth stages, 600 μL samples of the culture medium was collected and transferred to 1.5 mL microfuge tubes and centrifuged at 16,000 × g (5 min, 0°C). The supernatants were collected and placed on ice. For metabolite extraction from *T. marneffei* cells, cultures (20 mL) were rapidly cooled to 0°C by immersion of the culture flask in a dry ice/ethanol slurry (Saunders et al., 2011). Due to the inherent morphological differences between hyphal and yeast forms of *T. marneffei*, development of growth form-specific metabolite extraction procedures was required. For hyphal cells, aliquots of 1 × 10^8^ cell equivalents were centrifuged (16,000 × g, 15 min, 0°C), washed 3 times with equal volumes (~50 μL) of ice-cold PBS and divided into 4 technical replicates of 5 × 10^7^ cell equivalents. For yeast cells, aliquots of 1 × 10^8^ cell equivalents were harvested onto sterilized filter discs (Whatman, grade 1, 2.5 cm) at 0°C using a filter apparatus connected to a vacuum pump. Samples were washed thrice with equal volumes of ice-cold PBS (~20 μL) and the PBS was aspirated by vacuum. The washed discs were divided into quarters (~2.5 × 10^7^ cell equivalents each) using a sterile blade and cells were scraped into pre-chilled microfuge tubes.

Metabolite extraction was performed by addition of an organic solvent system (chloroform:methanol:water, 1:3:1 v/v; 250 and 500 μL for hyphal and yeast samples, respectively) (Saunders et al., 2011). In both cases samples contained 1 nmol *scyllo*-inositol (Sigma) as an internal standard. At this point yeast cell samples (2.5 × 10^7^ cell equivalents) were divided into 2 replicates of 1.25 × 10^7^ cell equivalents each. All samples were then subjected to 3 rounds of pulse vortexing, thawing sonication (5 min), and freeze-thaw (using liquid nitrogen). Cell lysis was verified by light microscopy and samples were incubated at 60°C for at least 30 min. Samples were centrifuged (16,000 × g, 5 min, 25°C) and the resultant supernatant (containing extracted polar and apolar metabolites) was transferred to a new microfuge tube. Biphasic partitioning of the polar and apolar metabolites was achieved by addition of H_2_O (final ratio chloroform:methanol:water, 1:3:3 v/v). The samples were then processed and analyzed by GC-MS using established protocols, as described briefly below.

### Analysis of polar phase metabolites

Polar phase metabolites were transferred to GC-MS vial inserts, dried using a rotary vacuum centrifuge, and washed twice with methanol. Dried extracts were resuspended in 20 μL of methoxyamine hydrochloride (Sigma, 20 mg/mL in pyridine), incubated at RT for 16 h and derivitized using 20 μL bis(trimethylsilyl)-trifluoroacetamide/1% trimethylchlorosilane (BSTFA/TCMS, Pierce). Samples were analyzed by GC-MS under conditions described previously (Saunders et al., 2011). Data was analyzed using ChemStation software (version D.01.02.16, Agilent Technologies). Metabolites were identified and quantified by comparison to authentic standards. As hyphal and yeast forms are morphologically different, the peak values for individual metabolites were normalized through division by the median of the entire (identified) data set of that cell type.

### Analysis of apolar phase metabolites

Apolar extracts were dried in a glass capillary, washed twice with methanol, and free fatty acids and lipid-containing molecules were derived by methanolysis, as described previously (Ramakrishnan et al., 2012). Briefly, samples were resuspended in 50 mL 0.5 N methanolic hydrochloric acid (Supelco), sealed under slight vacuum, and incubated at 90°C for 16 h. Cooled samples were neutralized with 10 mL pyridine, dried in a GC-MS vial insert, derived using 25 mL of N-methyl-N-(trimethylsilyl)-trifluoroacetamide/trimethylchlorosilane (MSTFA/TMCS, Pierce) and analyzed by GC-MS as described previously (Ramakrishnan et al., 2012). Data was analyzed using ChemStation software (version D.01.02.16, Agilent Technologies). Metabolites were identified and quantified by comparison to authentic standards.

### Labeling of *T. marneffei* hyphae and yeast stages with ^13^C-glucose

*T. marneffei* cells were grown in HI medium containing 6 mM glucose as major carbon source. Cultures were supplemented with U-^13^C-glucose [6 mM]_fin_ (Cambridge Isotope Laboratories) when cells reached mid-log phase. An aliquot of the culture medium was harvested at the indicated time-points following initiation of labeling and metabolites extracted as described above. The level of 13C-labelling of individual metabolites was quantified as a percentage of the metabolite pool containing one or more^13^C atoms after correction for natural abundance. The mass isotopomer distributions of individual metabolites were corrected for the natural abundance of ^13^C-atoms in an unlabeled equivalent in both the metabolite and the derivitization reagent (Zamboni and Sauer, 2009). Aliquots of starter medium from each experiment were also derivitized and analyzed by GC-MS (as above) to ensure the metabolite availability was consistent between experiments.

### Macrophage isolation and infection

THP-1 human macrophages were grown in RPMI (Sigma) containing 10% fetal bovine serum, 2 mM L-glutamine and 1 × penicillin-streptomycin solution. Macrophages (1 × 10^5^) were seeded into 6-well microtitre plates containing sterile coverslips and incubated overnight at 37°C in RPMI medium. Macrophages were then activated with the addition of lipopolysaccharide (LPS, 0.1 μg/mL), differentiated with phorbol 12-myristate 13-acetate, and incubated for a further 24 h at 37°C. Macrophages were then infected with 1 × 10^6^ *T. marneffei* conidia for 2 h to allow phagocytosis of conidia. Coverslips were then gently washed twice with PBS to remove free unengulfed conidia, 2 mL of fresh medium containing a range of NaFAc concentrations were added and macrophages were incubated for 24 h.

### Microscopy

*T. marneffei* infected THP-1 macrophages adhered on coverslips were fixed with 4% paraformaldehyde in PME buffer (50 mM PIPES, 1 mM MgSO_4_, 5 mM EGTA) for 30 min at room temperature and washed twice with PBS. Cells were then stained with fluorescent brightener 28 (Calcofluor, CAL, [0.014 mg/mL]_fin_) in Tween 80 (Sigma-Aldrich) and inspected microscopically. The number of *T. marneffei* conidia, germlings, hyphae and yeast cells in 100 macrophages were recorded. Three biological replicates were performed. Slides were examined using differential interference contrast (DIC) and fluorescent optics on a Reichart Jung Polyvar II microscope.

## Results

### Carbon source utilization and growth by hyphal and yeast cell types

**Figure 1:**
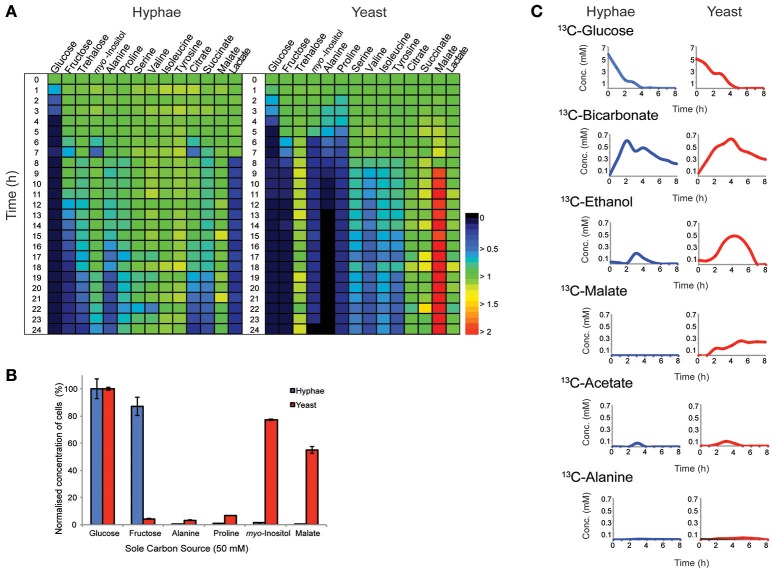
Consumption and production of metabolit by *T. marneffei* hyphal and yeast cells. **(A)** The abundance of metabolites detected in the growth medium of hyphal and yeast cells over 24 h, detected by GC-MS. Key metabolite levels are presented relative to abundances at time zero (*n* = 3). Changes in metabolite level are presented as a color gradient ranging from depletion (0) to secretion (2) relative to time zero (1). **(B)** Hyphal and yeast cell growth rates when cultivated in medium containing selected carbon sources. Growth was determined by optical density and normalized to the cell density measured when medium contained glucose as the major carbon source. Error bars represent the standard error of the mean (SEM) where *n* = 3. **(C)** Metabolic fate of U-^13^C-glucose in *T. marneffei* hyphal and yeast cells. Aliquots of the growth medium were sampled hourly for the first 8 h and then again at 24 h and analyzed by ^13^C-NMR and the average variance was <5%.

*T. marneffei* grows as multinucleated hyphal cells at 25°C and uninucleate yeast cells at 37°C *in vitro* and in the host. Both hyphal and yeast cells undergo exponential growth when suspended in fresh medium before reaching stationary growth, although the maximum rates of biomass accumulation differ between the two cell types (Figure S1). Based on labeling of hyphal and yeast lipids with U-^13^C-glucose and measurement of the turnover of bulk surface glycolipids, we estimated that hyphal cells accumulate biomass at a rate that is 2-fold faster than in the yeast cells during exponential growth (Figure S2). To determine whether both cell types exhibit similar carbon source utilization during exponential growth, *T. marneffei* hyphal and yeast cells were cultivated in complex HI medium and changes in potential carbon sources in the medium over 24 h monitored by GC-MS (Figure 1A, Tables S1, S2). While both cell types preferentially utilized glucose, significant cell type-specific differences were observed in the extent to which other carbon sources were utilized following depletion of glucose. Specifically, hyphal cells switched to using lactate and then fructose after glucose was depleted, followed by select amino acid (alanine), disaccharides (trehalose) and organic acids (citrate and succinate; Figure 1A). In contrast, yeast cells depleted glucose at a slower rate than hyphal cells, but then switched to simultaneously utilizing fructose, alanine, proline, and *myo*-inositol. This was followed by the depletion of several additional amino acids. Interestingly, the depletion of glucose and switch to alternative carbon sources in yeast cells was associated with secretion of malate and succinate, as well as low levels of trehalose, which likely represent overflow metabolites of the TCA cycle and carbohydrate storage, respectively, in these cells.

To confirm whether these cell type-specific differences in carbon utilization are functionally important, *T. marneffei* hyphal and yeast cells were cultured in defined liquid medium containing different sugars, amino acids and organic acids as sole carbon sources (Figure 1B). Consistent with the extracellular metabolite foot-printing analyses, hyphal cells grew rapidly in medium containing glucose or fructose, but not in medium containing *myo*-inositol or malate. In contrast, yeast cells grew poorly on fructose, but were able to utilize *myo*-inositol and malate as carbon sources. Neither cell type grew well on amino acids (alanine and proline) when these were provided as the sole carbon sources, although yeast cells did show some biomass accumulation under these conditions (Figure 1B). Collectively, these studies highlight marked differences in expression and/or regulation of nutrient transporters (fructose/*myo*-inositol) and/or metabolic pathways involved in the catabolism of specific carbon sources in hyphal and yeast cells.

### Cell type-specific glucose metabolism indicates fundamental differences in metabolic capabilities of hyphal and yeast cells

Hyphal and yeast cells were cultured in HI medium containing U-^13^C-glucose and changes in the levels of different ^13^C-labeled metabolites monitored by ^13^C- nuclear magnetic resonance spectroscopy (NMR) to define the basis of metabolic differences in these cell types. As expected, both cell types rapidly utilized the U-^13^C-glucose, depleting this carbon source within 4 or 5 h in hyphal and yeast cultures, respectively (Figure 1C). The major secreted end-product of both cell types was ^13^C-bicarbonate (~10% of total glucose consumed) indicating operation of the mitochondrial TCA cycle and/or other CO_2_ generating pathways, such as the pentose phosphate pathway. Both hyphal and yeast cells also secreted small amounts of ^13^C-ethanol and ^13^C-acetate during glucose catabolism, while yeast additionally secreted, ^13^C-malate and low amounts of ^13^C-alanine. The low rates of secretion of metabolic end-products overall (<15% of total ^13^C-glucose consumed) is consistent with most of the consumed glucose being used for biomass accumulation.

**Figure 2:**
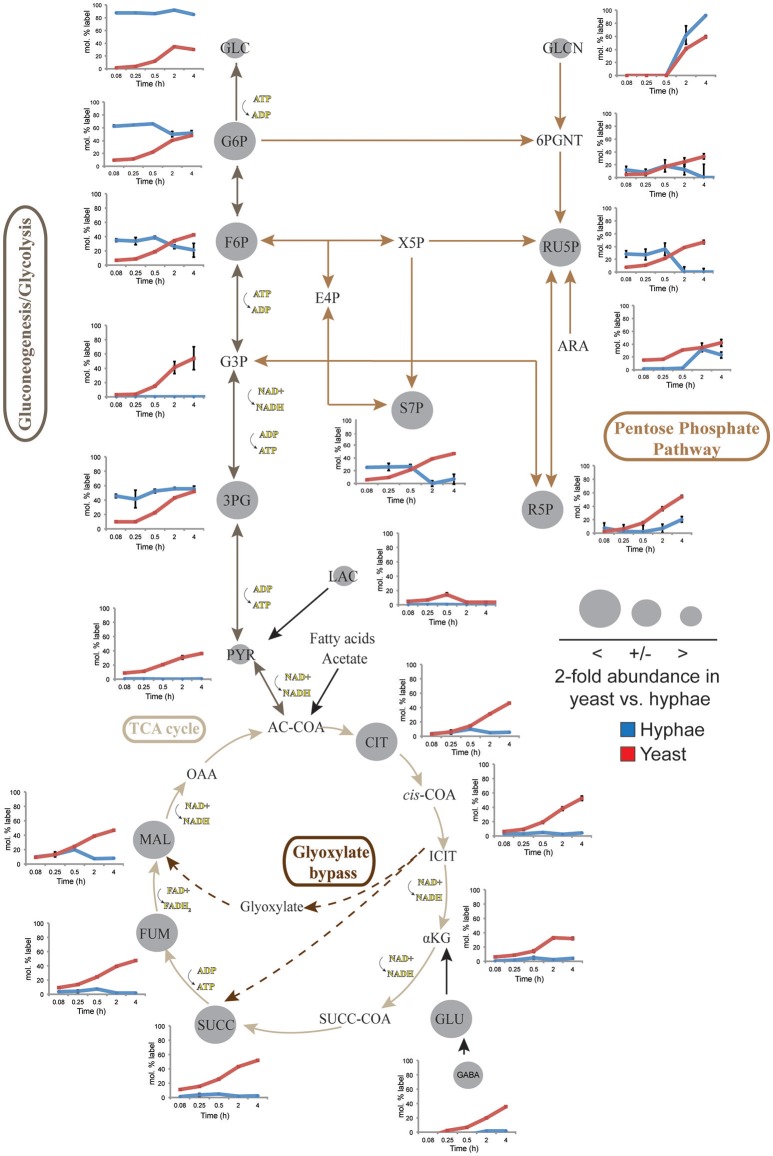
^13^C-labeling of hyphae and yeast central carbon metabolism intermediates. *T. marneffei* hyphae (blue lines) or yeast (red lines) were cultivated in HI medium containing ^13^C-glucose (6 mM) as sole carbon source. Cells were sampled at indicated time points and intracellular metabolite pools analyzed by GC-MS. ^13^C-enrichment in individual metabolites and key pathways in central carbon metabolism are shown. ^13^C-label incorporation is presented as the fraction of molecules containing one or more ^13^C atoms (after correction for ^13^C-natural abundance) (*n* = 3–4). Gray circles denote the 2-fold abundance of each metabolite detected in yeast cells relative to hyphal cells. Ac-CoA, acetyl-CoA; ADP, adenosine diphosphate; Ara, arabinose; ATP, adenosine triphosphate; Cit, citrate; E4P, erythrose 4-phosphate; F6P, fructose 6-phosphate; Fum fumarate; Glcn, gluconate; 6PGnt, 6-phospho-D-gluconate; Glc, glucose; G6P, glucose 6-phosphate; G3P, glyceraldehyde 3-phosphate; 3PG, 3-phosphoglycerate; iCit, isocitrate; αKG, ketoglutarate; Lac, lactate; Mal, α- malate; NAD, nicotinamide adenine dinucleotide; NADH, nicotinamide adenine dinucleotide reduced; OAA, oxaloacetate; Pyr, pyruvate; R5P, ribose 5-phosphate; Ru5P, ribulose 5-phosphate; S7P, *sedo*-heptulose 7-phosphate; Succ, succinate; Succ-CoA, succinyl-CoA; TCA, Tricarboxylic acid cycle; X5P, xylulose 5-phosphate.

**Figure 3:**
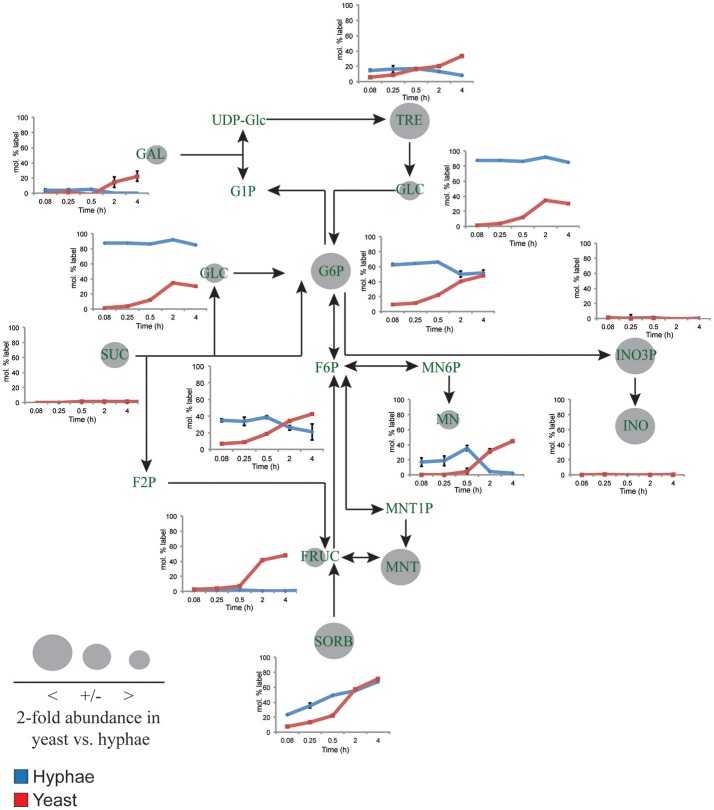
^13^C-labeling of hyphal and yeast storage sugar metabolism. *T. marneffei* hyphae (blue lines) or yeast (red lines) were cultivated in HI medium containing ^13^C-glucose (6 mM) as sole carbon source. Cells were sampled at indicated time points and intracellular metabolite pools analyzed by GC-MS. ^13^C-enrichment in individual metabolites and key pathways in storage sugar metabolism are shown. ^13^C-label incorporation is presented as the fraction of molecules containing one or more ^13^C atom (after correction for ^13^C-natural abundance) (*n* = 3–4). Gray circles denote the 2-fold abundance of each metabolite detected in yeast cells relative to hyphal cells. Fruc, Fructose; F2P, fructose 2-phosphate; F6P, fructose 6-phosphate; Gal, galactose; Glc, glucose; G1P, α-glucose 1-phosphate; G6P, glucose 6-phosphate; INO, *myo*-inositol; INO3P, *myo*-inositol 3-phosphate; UDP-Glc, UDP-glucose; Mnt, mannitol; Mnt1P, mannitol 1-phosphate; Mn6P, mannose 6-phosphate; Mn, mannose; Sorb, sorbitol; Suc, sucrose; Tre, trehalose.

To define further the metabolic pathways that were active in hyphal and yeast cells during glucose catabolism, polar metabolites were extracted from metabolically quenched cells and analyzed by GC-MS (Figure 2, Figures S3–S5, Tables S3, S4). ^13^C-glucose was rapidly incorporated into glycolytic intermediates in hyphal cells, reaching isotopic steady state within 5 min (Figure 2). Sequential step-downs in maximum ^13^C-enrichment in glucose-6-phosphate (Glc6P) and fructose-6-phosphate (Fru6P), compared to intracellular pools of ^13^C-glucose were observed, consistent with low level input of ‘unlabeled’ hexose-phosphate from catabolism and turnover of intracellular pools of trehalose-6-P and mannitol (Figure 3). Intermediates in the pentose phosphate pathway were also rapidly labeled, although at lower levels than in Glc6P. Strikingly, ^13^C-labeling of key TCA cycle intermediates (citrate, α-ketoglutarate, succinate, malate, and fumarate) in hyphal cells was generally low and decreased after 0.5 h indicating relatively low flux of glycolytic pyruvate and malate into the mitochondrion. The relatively high rate of ^13^C-bicarbonate production by this stage (20% of total glucose uptake), is therefore likely to reflect flux through the pentose phosphate pathway or conversion of pyruvate to ethanol (with regeneration of NAD^+^), rather than oxidative phosphorylation.

While yeast also rapidly consumed ^13^C-glucose, the kinetics of labeling of glycolytic intermediates was much slower than in hyphal cells (Figure 2) reaching isotopic equilibrium only after 4 h (Figure 2). Yeast cells contain much larger intracellular pools of hexose-phosphates than hyphal cells and also accumulate very high levels of trehalose and mannitol (Figure 3). The continuous turnover of these low molecular weight carbohydrates would account for the dilution of ^13^C-labeled Glc6P and the initial low level of labeling of all glycolytic and pentose phosphate pathway intermediates. The extent to which yeast cells produce other carbohydrate reserves, such as glycogen, was not investigated in this study but could also contribute to the delayed kinetics of labeling of central carbon intermediates. As the pools of trehalose and mannitol reached isotopic equilibrium with exogenous ^13^C-glucose, the level of labeling of intracellular down-stream intermediates increased rapidly, consistent with trehalose and mannitol having important roles as metabolic buffers. In marked contrast to hyphal cells, ^13^C-enrichment in pyruvate and TCA cycle intermediates in yeast, closely matched the labeling in the glycolytic intermediates (Figure 2). Increased flux of pyruvate into the TCA cycle was further supported by the finding that yeast cells contain elevated levels of TCA cycle intermediates (3- to 7-fold higher than in hyphae) and mass isotopomer distribution (MID) analysis of the yeast cell TCA intermediates. The latter confirmed the operation of a canonical TCA cycle through incorporation of ^13^C_2_ units from glycolysis-derived ^13^C_2_-labeled acetyl-CoA (Figure S6). Specifically, +2 and +4 (and +6 in citrate) isotopomers were detected in all TCA intermediates after 4 h, indicative of operation of a canonical TCA cycle and complete oxidation of acetyl-CoA. In addition, the presence of +3 (and +5 in citrate) isotopomers in all intermediates indicated significant flux through anapleurotic incorporation from carboxylation of phosphenolpyruvate or pyruvate to oxaloacetate (via phosphenolpyruvate carboxykinase, PMAA_032350 and pyruvate carboxylase, PMAA_094260). Taken together these analyses indicate that yeast cells up-regulate the extent to which glucose is catabolized in the TCA cycle and their dependence on oxidative phosphorylation for ATP synthesis. They also suggest a high level of cycling of glucose through trehalose and mannitol synthesis and turnover, which may constitute a mechanism for storing and buffering glucose flux. In contrast, hyphal cells primarily catabolize glucose via glycolysis with minimal flux of pyruvate or other glucose-derived anabolic intermediates into the TCA cycle.

### The functional importance of elevated TCA cycle catabolism in yeast cells

**Figure 4:**
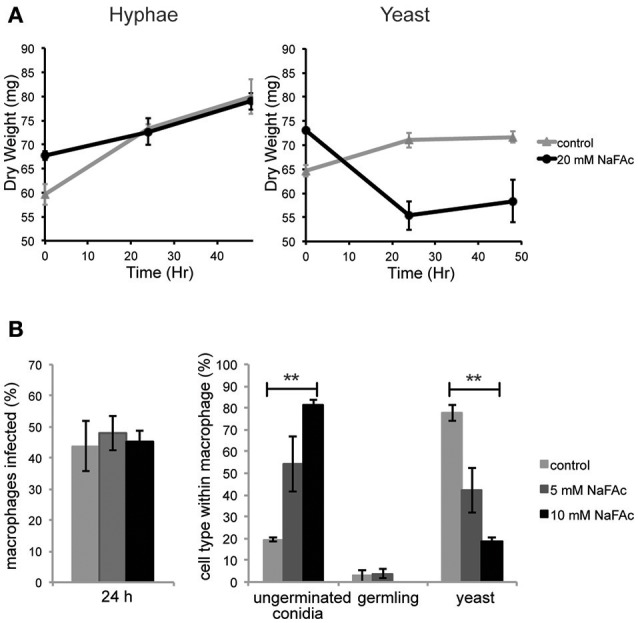
An active TCA cycle is important for *in vitro* and intracellular growth of *T. marneffei* yeast cells. **(A)** *T. marneffei* hyphal and yeast were cultivated in minimal medium in the presence or absence of NaFAc (20 mM). Growth (increase in dry weight) was monitored at 24 and 48 h (*n* = 3). **(B)** THP-1 macrophages were infected with *T. marneffei* conidia in the presence or absence of 5 or 10 mM NaFAc. Panels show the efficiency of conidia invasion of macrophages after 24 h; the proportion of conidia that generate to yeast cell in the macrophage phagosome; and growth of the germinated yeast cells. Conidia and yeast cells were monitored microscopically. Error bars represent the standard error of the mean (^**^denotes *p* < 0.005) of three biological repeats.

To investigate the role of elevated mitochondrial metabolism in yeast cells, both yeast and hyphal cells were grown in minimal medium containing glucose as the major carbon source and then treated with NaFAc. FAc is converted to fluoroacetyl-CoA and fluorocitrate *in vivo* and is a potent and specific inhibitor of the TCA cycle enzyme, aconitase (Armitt et al., 1976). Hyphal cells grew at the same rate as control-treated cells in the presence of NaFAc, indicating that TCA cycle activity is not required for optimal growth. In marked contrast, growth of yeast cells was greatly reduced in the presence of 20 mM NaFAc (Figure 4A), indicating a strong dependence on the TCA cycle for energy metabolism and/or toxicity associated with citrate accumulation due to inhibition of citrate conversion to isocitrate. Inhibition of TCA cycle activity also resulted in attenuation of fungal growth inside THP-1 macrophages. Specifically, while phagocytosis of conidia by macrophages was unaffected by increasing concentrations of NaFAc (5, 10 mM), conidial germination and conversion to yeast cells was markedly reduced in the presence of 10 mM NaFAc (Figure 4B). THP-1 macrophage viability remained >98% in all treatments, reflecting the high glycolytic metabolism of these host cells. These results suggest that the TCA cycle and oxidative phosphorylation has a non-redundant role in the energy metabolism of yeast cells both *in vitro* and during infection of host cells.

### Rewiring of carbohydrate reserves and *myo*-inositol metabolism in yeast cells

Yeast cells accumulated 6- to 10-fold higher levels of the compatible solutes mannitol and trehalose compared to hyphal cells. These carbohydrates can be used as short-term energy reserves and have also been shown to have a role in regulating glycolytic flux and redox levels under fluctuating nutrientconditions. Both trehalose and mannitol were labeled in ^13^C-glucose-fed yeast cells, consistent with constitutive turnover of these carbohydrates under glucose-replete conditions. Significant enrichment of ^13^C was also seen in the trehalose and polyol pools of hyphal cells during early time points, but as the pool sizes of these reserves are much smaller than in yeast, the corresponding absolute rates of turnover are much lower (Figure 3). Interestingly, hyphal cells, but not yeast cells, were able to utilize exogenous trehalose as a carbon source (Figure 1A). These observations suggest that hyphal cells can either take up trehalose directly or secrete trehalases that break-down exogenous trehalose to glucose.

*m*yo-Inositol is an essential precursor for the synthesis of the bulk membrane lipid phosphatidylinositol (PI), as well as complex phosphoinositides and GPI anchors. *myo*-Inositol-phosphates also act as secondary messengers in many signaling pathways. Levels of both *myo*-inositol and its precursor *myo*-inositol-3-P are highly elevated in yeast cells (25- and 10-fold, respectively, compared to hyphal cells; Figure 3). Significantly, ^13^C-enrichment in *myo*-inositol and *myo*-inositol-3-phosphate following labeling of yeast with ^13^C-glucose was very low (<5% ^13^C-enrichment), indicating that these cells salvage most of the *myo*-inositol from the medium. This is consistent with the finding that yeast cells switch to using exogenous *myo*-inositol as a primary carbon source following depletion of glucose (Figure 1A).

### Cell type-specific changes in *De novo* amino acid

**Figure 5:**
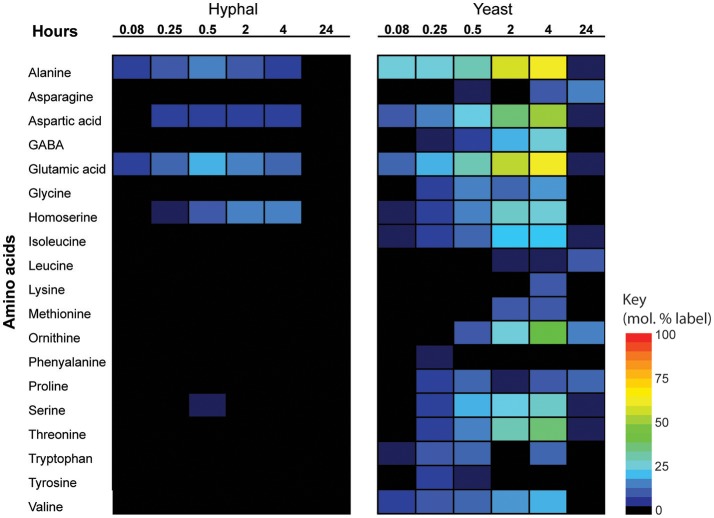
^13^C incorporation of amino acids in *T. marneffei* hyphal and yeast cells. After isotope addition, samples were harvested at the indicated time points over 24 h. ^13^C incorporation into each detected amino acid is shown as a heatmap where percent label incorporation is the percentage of molecules containing at least one ^13^C atom.

Significant cell type-specific differences were also observed in the extent to which *T. marneffei* hyphal and yeast cells synthesized different amino acids. Hyphal cells exhibited minimal levels of amino acid biosynthesis in complex medium, as shown by the very low to non-detectable levels of labeling of intracellular amino acid pools (Figure 5). ^13^C-enrichment in alanine, aspartic acid, glutamic acid and homoserine reflects the transamination reactions with key intermediates in central carbon metabolism (i.e., pyruvate, oxaloacetate, α-ketoglutarate and 3-phosphoglycerate). The absence of significant labeling in other amino acids indicated that these are primarily salvaged from the medium while glucose is present. In contrast, carbon skeletons derived from ^13^C-glucose were incorporated into most amino acids (albeit to various extents) in yeast cells (Figure 5). Strong labeling of alanine, aspartic acid, glutamic acid, and γ-amino butyric acid (GABA) was observed, reflecting increased TCA flux (Figures 2, 5). Significant ^13^C-enrichment in glycine, arginine/ornithine, serine/threonine, tryptophan and valine was also observed at early time points indicating elevated rates of *de novo* amino acid synthesis in yeast cells even under amino acid replete growth conditions (Figure 5). This suggests a coordinated switch to increased salvage and *de novo* synthesis in this cell type, indicative of a reprogramming of metabolism as a whole to becoming more “amino acid-centric” in yeast cells.

## Discussion

*T. marneffei* hyphal cells are thought to be versatile saprophytes that can grow on a wide range of substrates and exploit a broad range of environments (Andrianopoulos, 2002; Vanittanakom et al., 2006). While pathogenic yeast cells are commonly thought to be more fastidious they are capable of surviving and replicating within potentially nutrient-restricted niches in macrophages and other phagocytic cells. Yeast cells also need to employ robust mechanisms for withstanding a wide range of physical and chemical/oxidative stresses generated within host tissues. There is increasing evidence that dimorphic switching between hyphal and yeast cell types in other fungi extends the host/environmental range of these pathogens, but few studies have investigated changes in metabolism that occur during switching and how these changes define the capacity to colonize specific niches. Here we show that hyphal and yeast cells display distinct and significant differences in their capacity to utilize different carbon sources and their dependence on major pathways of central carbon metabolism. These differences highlight metabolic features that are likely integral to the maintenance of *T. marneffei* hyphal cell growth in the environment and the survival of yeast cells within host macrophages. While there are no similar unbiased metabolomic studies geared to understanding the core differences between hyphal and yeast cell types in other dimorphic fungal pathogens, the metabolic pathways identified here show some overlap with what is predicted from transcriptional profiling studies in other dimorphic fungal pathogens such as *H. capsulatum* and *C. albicans*, as well as *T. marneffei* (Hwang et al., 2003; Lorenz et al., 2004; Pasricha et al., 2013). It is important to note that some of the pathways identified in this study that differentiate *T. marneffei* hyphal and yeast cell metabolism and are predicted to be host niche adaptive were identified during yeast cell growth *in vitro*. As such it is likely that yeast cells are metabolically pre-adapted, or hard-wired, for growth in the host requiring fewer specific pathogen responses to the intracellular host environment.

**Figure 6:**
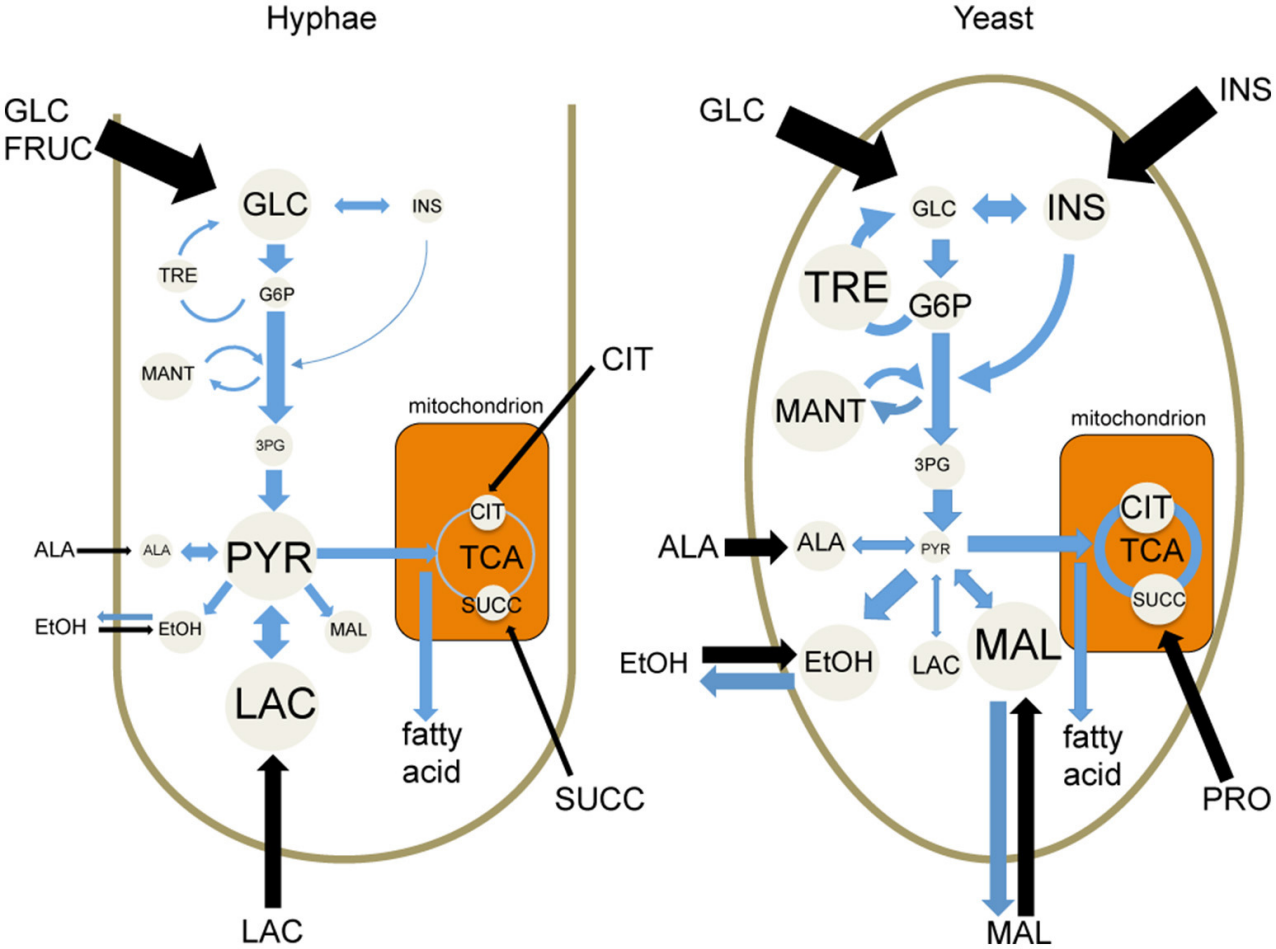
Cell type-specific metabolism of *T. marneffei*. Carbon utilization through the glycolytic pathway and the TCA cycle of *T. marneffei* hyphal and yeast cells is shown in cartoon form. Metabolite abundances are depicted by circle and font sizes, metabolic flux by the thickness of blue arrows and the relative level of metabolite uptake from exogenous medium is represented by the thickness of black arrows. Ala, Alanine; Cit, citrate; EtOH, ethanol; Fruc, fructose; Glc, glucose; G6P, glucose 6-phosphate; 3PG, 3-phosphoglycerate; Ins, inositol; Mal, malate; Mant, mannitol; Pyr, pyruvate; Succ, succinate; TCA, tricarboxylic acid cycle; Tre, trehalose.

While both *T. marneffei* hyphal and yeast cell types preferentially utilize glucose as a carbon source, significant differences were observed in the extent to which these cell types were dependent on aerobic glycolysis or mitochondrial metabolism. Specifically, hyphal cells exhibited a predominantly fermentative metabolism, catabolizing glucose via glycolysis and the pentose phosphate pathway to generate ethanol and CO_2_, with minimal catabolism of pyruvate in the mitochondrial TCA cycle (Figure 6). In contrast, yeast cells appeared to further catabolize pyruvate in the mitochondrion, accounting for the continued production of CO_2_ by this cell type, although yeast also secreted significant amounts of ethanol (Figure 6). Yeast cells also synthesized several amino acids that likely undergo transamination reactions to fuel the TCA cycle. This switch to a more energy efficient metabolism in yeast cells would reduce the requirement for exogenous glucose to sustain high growth rates and increase the capacity of this cell type to proliferate within macrophages and other phagocytic host cells. In support of this conclusion, we showed that pharmacological inhibition of *T. marneffei* yeast TCA cycle resulted in a marked reduction in yeast growth in culture and intracellular proliferation in macrophages. In contrast, growth of hyphal cells was largely unaffected by inhibition of the TCA cycle, consistent with increased dependence on fermentation.

The second difference in *T. marneffei* hyphal and yeast cell metabolism that may reflect the niche preferences of these cell types relates to their capacity to utilize carbon sources other than glucose. Hyphal cells were able to switch to using other sugars (i.e., fructose, trehalose) and lactate, but grew poorly in minimal medium containing amino acids or organic acids (succinate, citrate). This carbon source preference is likely to reflect the availability of different sugars in the as-yet unidentified environmental niches occupied by the *T. marneffei* hyphal form and/or limited capacity to quickly activate mitochondrial metabolic capacity. In contrast, yeast cells were able to rapidly switch from using glucose to other sugars including fructose and *myo*-inositol. Following depletion of these carbon sources, yeast increased utilization of a number of amino acids. Interestingly, only *myo*-inositol and malate sustained high rates of yeast growth when provided as sole carbon sources. A number of other fungal pathogens have been shown to utilize *myo*-inositol as a major carbon source *in vivo* and *myo*-inositol metabolism is essential for the virulence of *Cryptococcus neoformans* (Howard, 1973; Molina et al., 1999; Trinel et al., 1999; Fan et al., 2005; Xue et al., 2010; Vylkova et al., 2011). *myo*-Inositol is present at high concentrations in macrophages as well as in some tissues (i.e., brain) that may underlie fungal tropism for these cell types/tissues.

The third major difference between the *T. marneffei* yeast and hyphal cell types relates to synthesis and storage of carbohydrate reserves. While both hyphal and yeast cells synthesized trehalose and mannitol, these carbohydrates were present at much higher levels in yeast cells. These carbohydrates were labeled when log phase yeast cells were grown in the presence of ^13^C-glucose indicating constitutive turnover and a dynamic role in yeast metabolism. This was further supported by the delayed ^13^C-glucose labeling kinetics in glycolytic intermediates, consistent with initial cycling of Glc6P through the pools of trehalose/mannitol, before being channeled back into glycolysis and the pentose phosphate pathway. Trehalose and mannitol might therefore act as metabolic buffers in yeast, regulating the flux of glucose into major energy/redox generating pathways. It is possible that the synthesis and turnover of *myo*-inositol might fulfill a similar function under glucose-replete conditions.

Finally hyphal and yeast cells use sugars for the biosynthesis of abundant surface glycolipids but at different rates. The higher rate of turnover of mannolipids in hyphae likely reflects the faster growth rate of these cells compared to yeast. Similarly, the higher rate of glucose consumption and lower rate of overflow metabolism in hyphal cells indicate increased diversion of carbon skeletons into new biomass in these cells. While yeast cells growing either *in vitro* or inside host cells continue to replicate, the overall rate of macromolecule turnover appears to be lower than in hyphal cells. Hyphal cells thus appear to be geared toward efficient nutrient scavenging and rapid growth, a strategy that may allow them to out-compete other microbes in the absence of a traditional motile response. Conversely, yeast cell metabolism is optimized for slow growth in more diverse, but potentially nutrient-poor niches and the accumulation of compatible solutes (low molecular weight carbohydrates) that provide protections against a range of host directed cytotoxic insults.

This study defines a global exploration of metabolic differences between non-pathogenic and pathogenic *T. marneffei* cell-types from which a number of hypotheses about adaptation to particular niches for the two cell types have been devised. The differences in metabolism between the two cell types highlights the programmed changes during dimorphic switching that contribute to the establishment of macrophage infection by *T. marneffei* yeast cells. As temperature is a key driver of dimorphic switching, we can now examine *T. marneffei* mutant strains unable to undergo dimorphic switching, in order to uncouple the roles of morphology and temperature. This study has also laid down the foundation for employing metabolomic-profiling techniques on other pathogenic fungi. More targeted studies of the key aspects of observed metabolic switching will likely facilitate a deeper understanding of pathogenic virulence factors and, potentially, highlight rational therapeutic avenues to pursue against the pathogenic form.

## Author contributions

SP conceived and performed experiments, analyzed metabolomics data, and prepared manuscript. JM established metabolite protocols, performed experiments, analyzed metabolomics data and significantly contributed during experimental design and manuscript preparation. HC, JC performed experiments, analyzed data and contributed intellectually during manuscript preparation. KB contributed intellectually during experimental design and manuscript preparation. MM, AA conceived project, significantly contributed to experimental design, analysis, and manuscript preparation.

### Conflict of interest statement

The authors declare that the research was conducted in the absence of any commercial or financial relationships that could be construed as a potential conflict of interest.
