# Supplementary material for: Extensive Metabolic Remodeling Differentiates Non-pathogenic and Pathogenic Growth Forms of the Dimorphic Pathogen Talaromyces marneffei
Source: Front Cell Infect Microbiol. 2017 Aug 17;7:368. doi: 10.3389/fcimb.2017.00368 (PMC5563070; doi:10.3389/fcimb.2017.00368)
Supplement: Supplementary file 4 [file Table4.pdf]

Table S4. Normalised abundance of detected metabolites in hyphal and yeast cells.*

Metabolite	Hyphal normalised abundance (nmol)	Yeast normailsed abundance (nmol)	Fold difference of normalised abundance
**Glycolysis/Gluconeogensis intermediates**			
Pyruvic acid	68.39 (±30.50)	6.35 (±1.81)	**10.77**
Lactic acid	2.06 (±0.57)	0.46 (±0.09)	**4.46**
Glucose	0.20 (±0.13)	0.08 (±0.01)	2.40
Glucose 6-P	-	0.18 (±0.04)	∞
Fructose 6-P	-	0.17 (±0.03)	∞
aGP	1.11 (±0.31)	14.49 (±0.99)	**13.02**
bGP	12.84 (±6.51)	92.51 (±11.81)	**7.20**
3PGA	0.28 (±0.02)	0.99 (±0.09)	**3.54**
**TCA cycle intermediates**			
cis-Aconitate	0.05 (±0.01)	-	∞
Malic acid	0.23 (±0.04)	1.63 (±0.12)	**7.05**
Citric acid	0.98 (±0.26)	4.11 (±0.28)	**4.20**
Succinic acid	0.35 (±0.09)	1.36 (±0.42)	3.93
Fumaric acid	0.09 (±0.02)	0.27 (±0.05)	2.91
**Pentose Phosphate Pathway intermediates**			
Gluconic acid	0.05 (±0.02)	0.01 (±0.00)	**5.17**
Ribose 5-P	-	0.69 (±0.29)	∞
sedoHeptulose 7-P	-	0.45 (±0.14)	∞
Ribulose 5-P	-	0.01 (±0.00)	∞
Gluconic acid derv 1	0.14 (±0.07)	0.80 (±0.05)	5.73
Glucoinc acid derv 2	0.48 (±0.17)	0.57 (±0.04)	0.63
**Amino Acids**			
Lysine	19.06 (±3.04)	-	∞
Tryptophan	13.02 (±4.94)	-	∞
Tyrosine	6.56 (±2.91)	-	∞
Methionine	10.07 (±3.97)	1.01 (±0.87)	10.01
Isoleucine	2.42 (±0.71)	0.53 (±0.06)	**4.54**
Leucine	8.71 (±1.82)	2.15 (±0.18)	**4.05**
Phenylalaine	5.81 (±2.04)	1.57 (±0.39)	3.70
Valine	3.41 (±0.94)	1.38 (±0.13)	2.48
Asparagine	3.06 (±2.68)	1.30 (±0.39)	2.35
Glycine	1.22 (±0.34)	0.55 (±0.12)	2.22
Serine	2.20 (±0.64)	1.21 (±0.20)	1.82
Threonine	1.63 (±0.48)	1.02 (±0.16)	1.59
GABA	2.75 (±0.75)	2.59 (±0.57)	**1.06**
Homoserine	0.55 (±0.07)	3.17 (±0.90)	**5.79**
Ornithine	2.07 (±0.58)	7.58 (±0.90)	3.66
Glutamatic acid	8.69 (±1.36)	28.01 (±7.49)	**3.22**
Proline	0.55 (±0.19)	1.07 (±0.14)	1.95
Aspartic acid	15.48 (±4.02)	29.13 (±7.36)	1.88
Alanine	4.48 (±1.09)	8.06 (±0.59)	1.80
**Nucleotides and Nucleosides**			
Uracil	1.12 (±0.17)	3.73 (±0.32)	**3.32**
**Other sugers and suger phosphates**			
Galactose	0.34 (±0.13)	-	∞
Mannose	0.06 (±0.03)	-	∞
Fructose	7.65 (±2.73)	2.23 (±0.17)	3.42
myo-inositol	0.36 (±0.08)	9.09 (±2.05)	**24.91**
Mannitol/Sorbitol	0.07 (±0.02)	0.86 (±0.18)	**11.85**
myo-inositol 3-P	0.02 (±0.01)	0.14 (±0.12)	**7.93**
Trehalose	0.92 (±0.21)	5.93 (±0.92)	**6.46**
Erythrose	0.59 (±0.18)	2.40 (±1.07)	4.05
Lactulose	0.46 (±0.16)	0.58 (±0.08)	1.28
Sucrose	0.26 (±0.07)	0.29 (±0.04)	1.11
**Novel/unidentified glycosides**			
Unknown3	0.65 (±0.24)	-	∞
Disaccharide1	0.65 (±0.24)	-	∞
Unknown2	1.02 (±0.29)	0.19 (±0.03)	**5.24**
Unknown1	0.80 (±0.22)	0.35 (±0.03)	2.29
Pentitol	-	0.45 (±0.04)	∞
Pentose phosphate	-	0.45 (±0.07)	∞
Hexose phosphate	-	0.45 (±0.11)	∞
Unknown4	-	0.44 (±0.20)	∞
Disaccharide2	-	0.45 (±0.11)	∞

* Metabolites that are more abundant in hyphal cells (blue shading) and those that are more abundant in yeast cells (red shading) are indicated. Standard deviations (±), where n=4 are also shown. Undetected metabolites in a given cell type are denoted (-) and have infinite fold differences in abundance between cell-types (∞). Significant fold differences (bold) are based on T-tests, where $p < 0.001$.
Putative dervatives of gluconic acid are distinguishly labelled, derv 1 and derv 2.
