# Supplementary figures and images for: Extensive Metabolic Remodeling Differentiates Non-pathogenic and Pathogenic Growth Forms of the Dimorphic Pathogen Talaromyces marneffei
Source: Front Cell Infect Microbiol. 2017 Aug 17;7:368. doi: 10.3389/fcimb.2017.00368 (PMC5563070; doi:10.3389/fcimb.2017.00368)

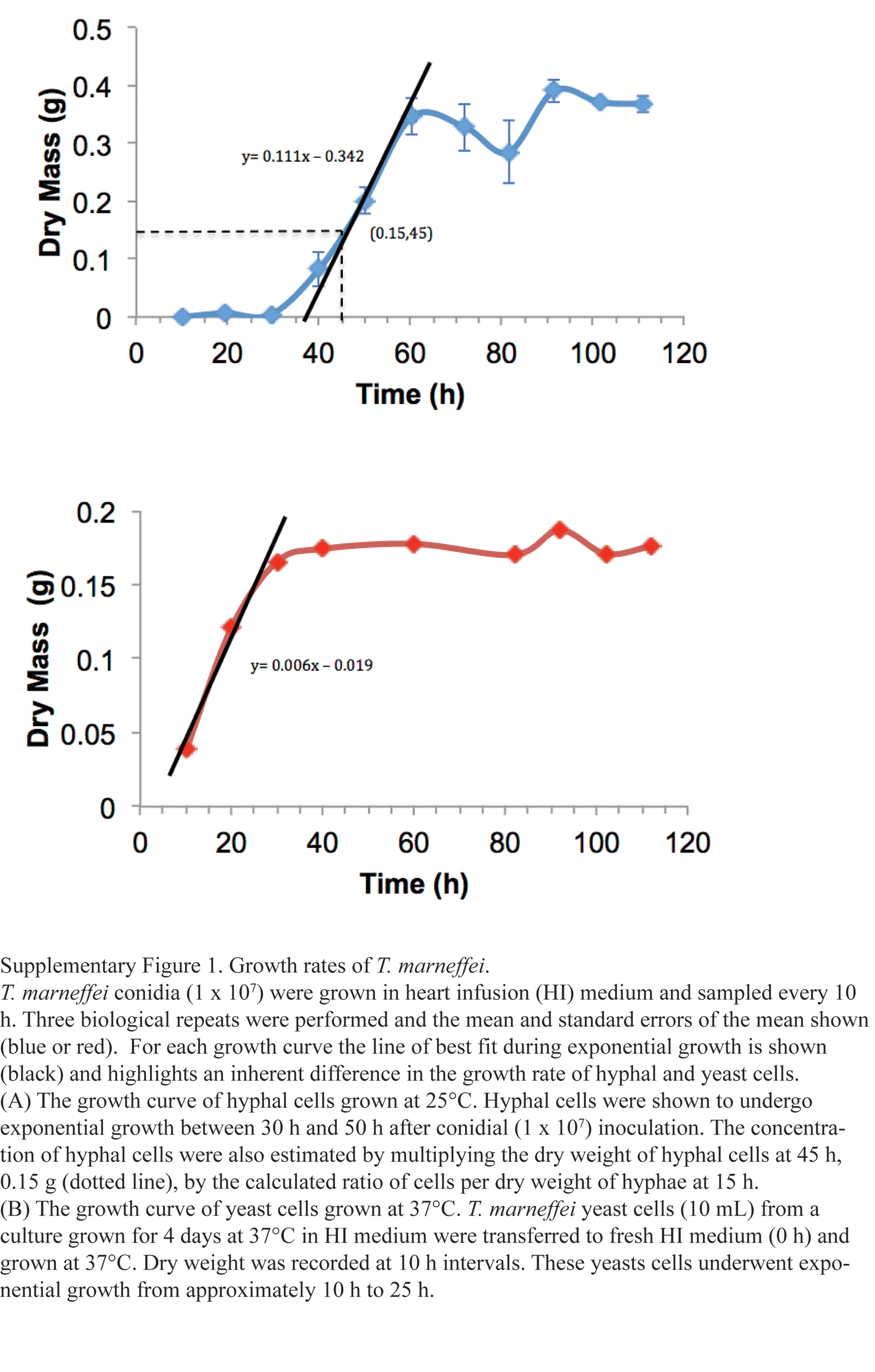

Supplement: Supplementary file 5 [file Image1.TIF]

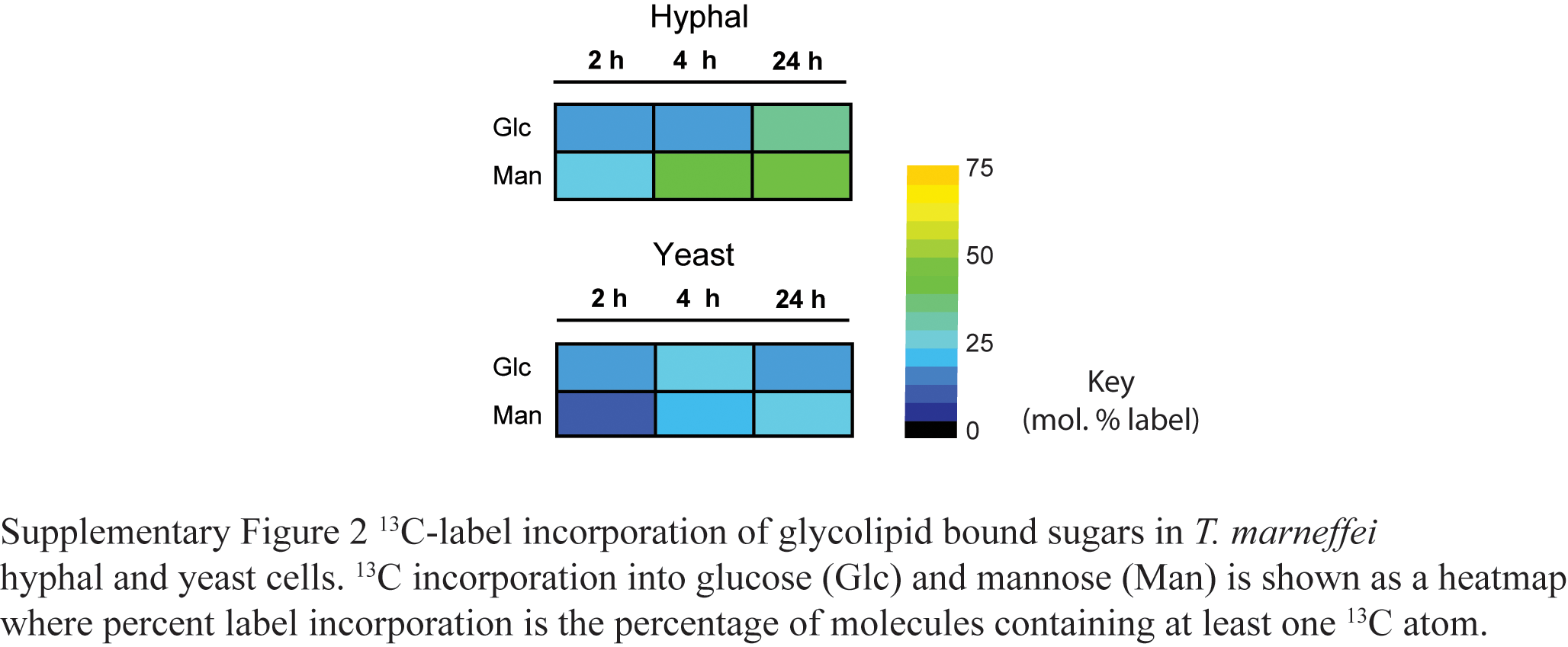

Supplement: Supplementary file 6 [file Image2.TIF]

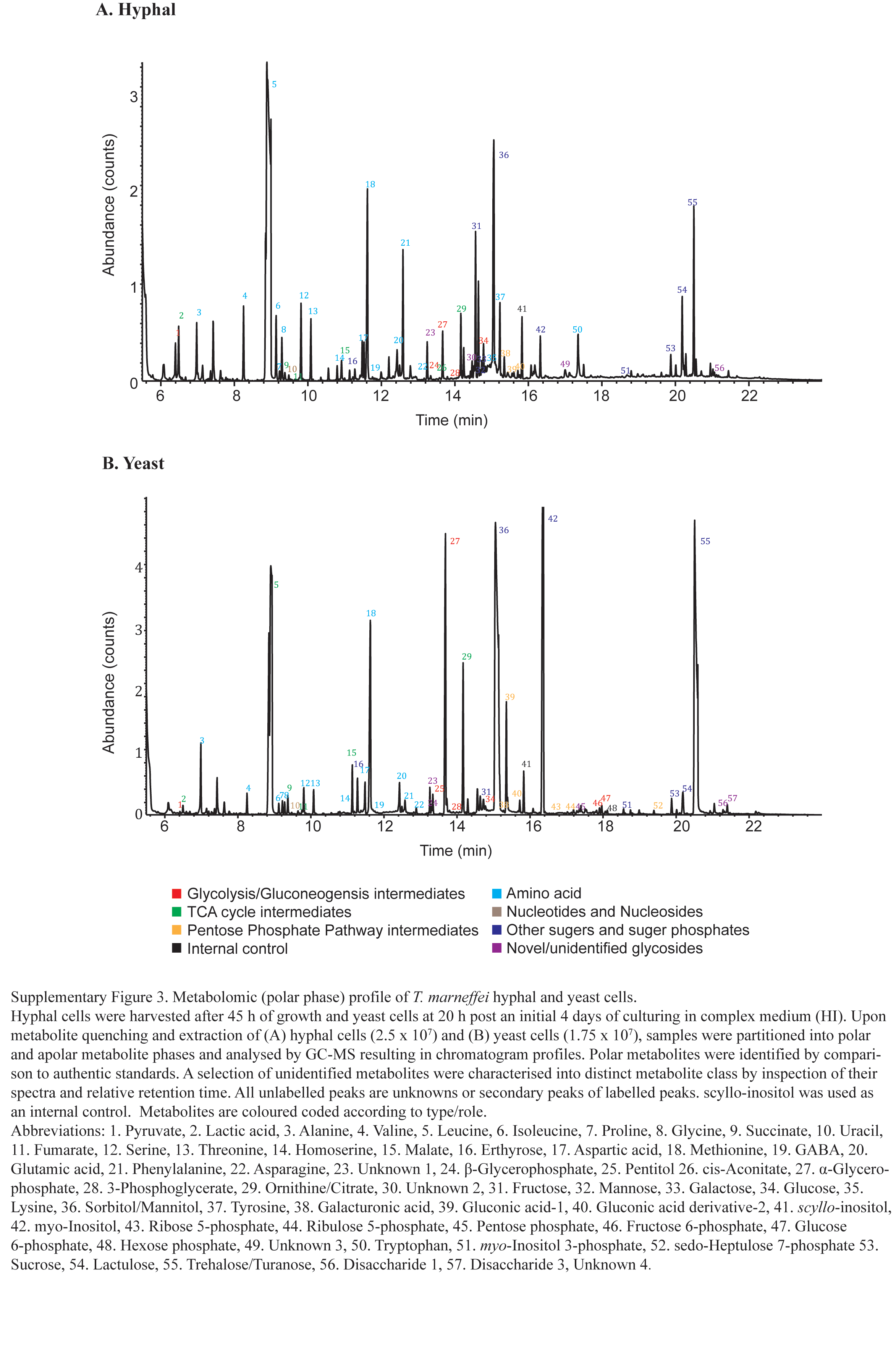

Supplement: Supplementary file 7 [file Image3.TIF]

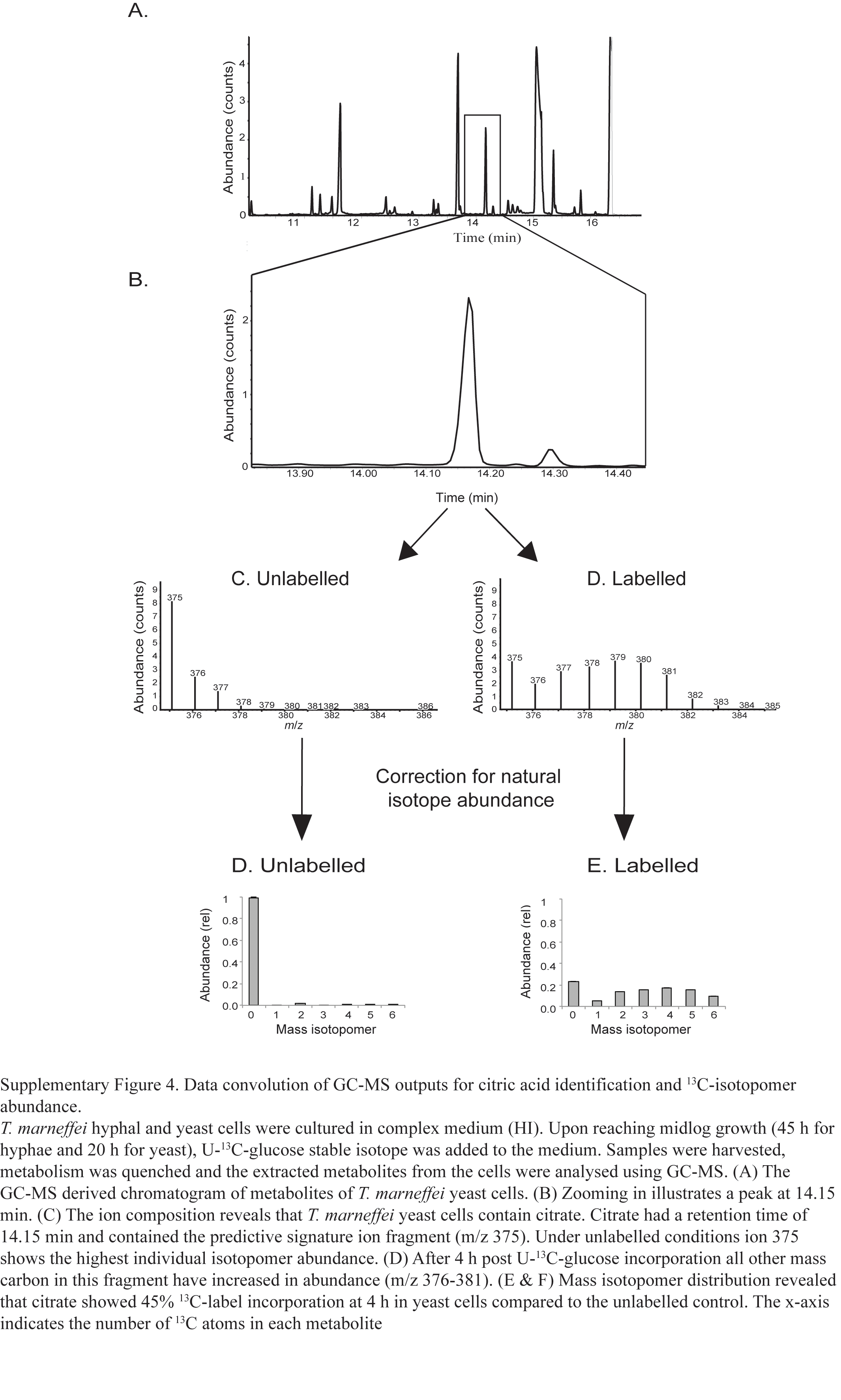

Supplement: Supplementary file 8 [file Image4.TIF]

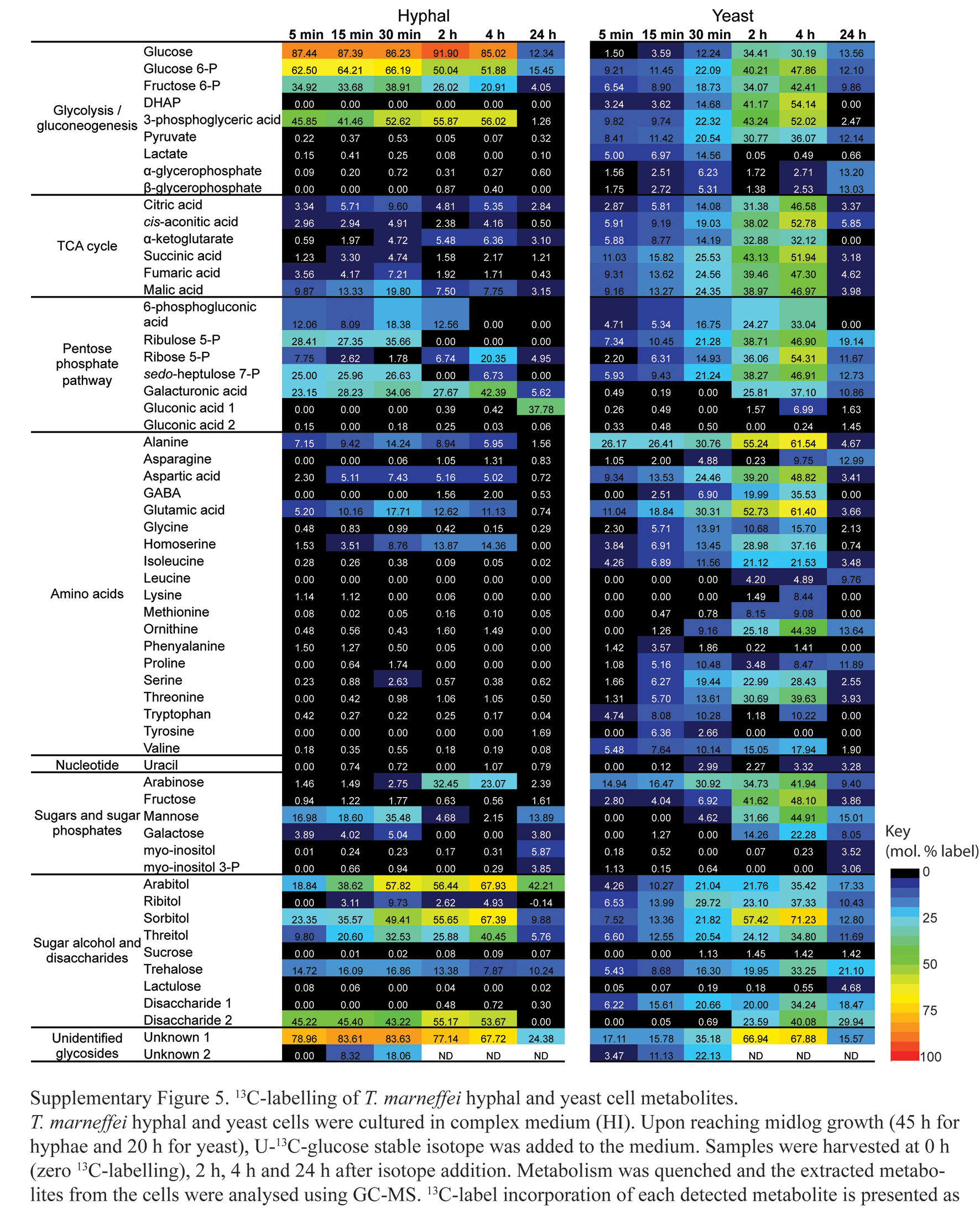

Supplement: Supplementary file 9 [file Image5.TIF]

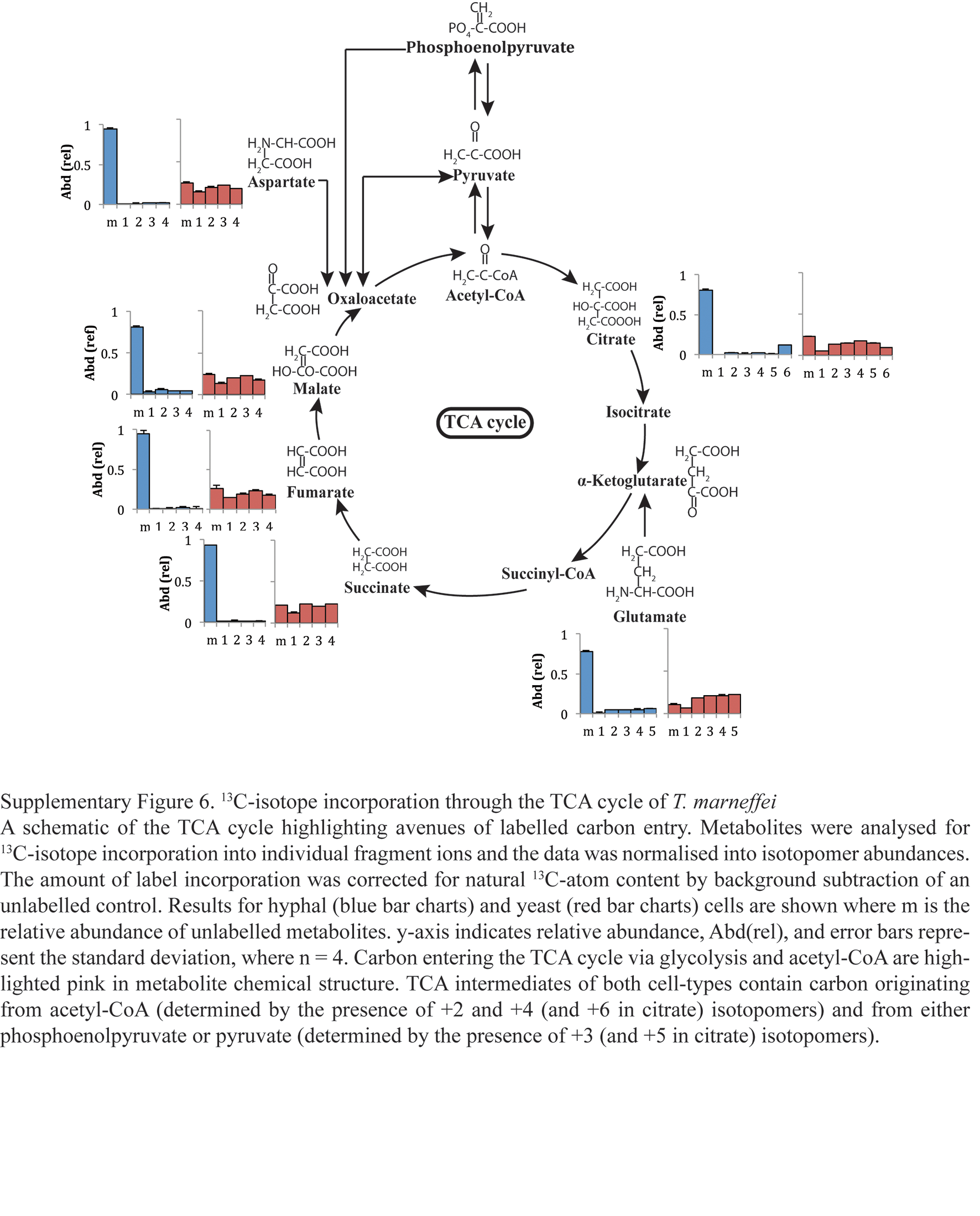

Supplement: Supplementary file 10 [file Image6.TIF]
